# Additive manufacturing in nano drug delivery systems

**DOI:** 10.1016/j.pscia.2024.100036

**Published:** 2024-02-21

**Authors:** Md. Habibur Rahman, Nilufar Yasmin Liza, Khan Rajib Hossain, Dipika Ramdas Kalambhe, Md. Abu Shyeed, Dilwar Hossain Noor

**Affiliations:** a Department of Applied Chemistry and Chemical Engineering, Islamic University, Kushtia, 7003, Bangladesh; b State Key Laboratory of Solid Lubrication, Lanzhou Institute of Chemical Physics, Chinese Academy of Sciences, Lanzhou, 730000, China; c Shanghai Institute of Materia Medica, Chinese Academy of Sciences, Shanghai, 200120, China; d University of Chinese Academy of Sciences, Beijing, China; e Department of Applied Chemistry and Chemical Engineering, University of Rajshahi, Rajshahi, 6205, Bangladesh

**Keywords:** 3D printing, Nano drug, Delivery, Hydrogels, Nanoparticles

## Abstract

The adoption of innovative mixing and fabrication technologies in the pharmaceutical industry has inspired research on nano-drugs and expanded the scope of study in the field. Researchers' interest in the recently discovered drug delivery nanoparticles has increased significantly. This interest is especially seen in the specific preparation of nanoparticles as drug carriers through the use of three-dimensional (3D) printing, a modern additive manufacturing (AM) technology. The benefits of 3D printing, especially at the nanoscale, make it an innovative technology that could revolutionize the pharmaceutical and regenerative medicine industries. The laborious creation of intricate structures made possible by nanoscale 3D printing brings up the possibility of developing nanomedicine and producing functioning tissues and organs. The uses of AM in nano drug delivery systems (NDDS) are highlighted in this study, with a focus on how it can improve drug release kinetics, enhance therapeutic efficacy, and minimize side effects. A new era of personalized medicine has begun with the development of patient-specific formulations made possible by the customization capabilities of 3D printing. This review discusses the various uses of AM in NDDS while addressing issues including scalability, biocompatibility, and regulatory concerns. This review highlights the developing integration between AM and nanotechnology in medicine delivery and discusses ongoing research activities and possible solutions. As this innovative technology develops further, it has the potential to completely change the pharmaceutical development field by providing fresh approaches to the complex problems of contemporary healthcare and advancing the ideas behind drug delivery.

## Highlights

• 3D printing nano drug is a viable option to improve biomedical, tissue engineering, implants, and transdermal drug delivery.
• Additive Manufacturing (AM) is a suitable technique for fabricating nano drugs.
• Fundamental concepts for 3D printers used in pharmaceutical formulations.
• 3D printing is a highly efficient and effective method for producing low-cost medicine.

## Graphical abstract

**Figure:**
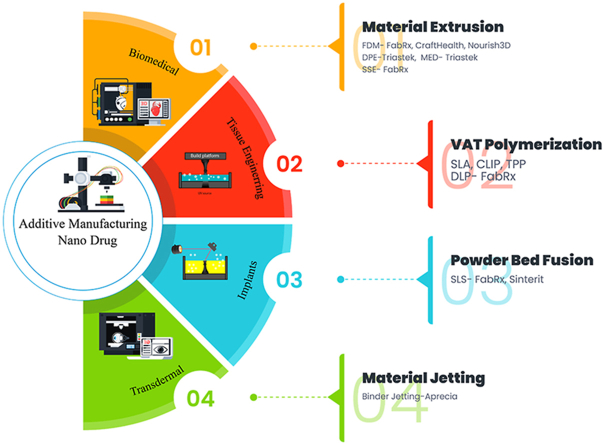

## Abbreviation list

3D: Three-Dimensional
3DP: 3D Printing
AM: Additive Manufacturing
API: Active Pharmaceutical Molecule/Ingredients
ASD: Amorphous Solid Dispersions
CRDDS: Controlled Drug Delivery System
DLP: Digital Light Processing
FDA: Food and Drug Administration
GMP: Good Manufacturing Practice
HME: Hot Melt Extrusion
MED: Melt Extrusion Deposition Technology
MJ/Polyjet: Material Jetting
MN: Microneedle
NDDS: Nano Drug Delivery Systems
nm: Nanometer
SLA: Stereolithography
SLS/SLM: Selective Laser Sintering Technology

## 1 Introduction

Since Aprecia's three-dimensional (3D)-printed drug Spritam® (levetiracetam tablets) received marketing approval from the US Food and Drug Administration (FDA) in 2015, the interest of the pharmaceutical industry in 3D printing has steadily increased [1]. Several physicochemical and biological properties of lead compounds are geared toward binding to targets. However, formulation scientists have garnered considerable attention for developing patient-centered products with their unique technical aspects. 3D printing, often known as 3DP, is widely regarded as the most game-changing and potentially lucrative technology in the pharmaceutical and biomedical industries, among any of the more recent breakthroughs. Pharmaceutical manufacturers are getting increasingly interested in 3D printing due to its potential advantages in producing pharmaceutical formulations. 3D printing technology has digital attributes and is expected to achieve significant breakthroughs in the commercial production of blockbuster products or personalized drug delivery [2].

Additive manufacturing (including 3D printing) technologies can provide many potential benefits to pharmaceutical manufacturers, such as solving the solubilization of poorly soluble drugs by developing simpler drug formulations and utilizing more efficient continuous production methods for the production of complex formulations and bioavailability, among other issues. The ability of 3D printing technology to accommodate larger-scale clinical and commercial requirements, its flexible production capabilities, and its process analysis technology give it significant potential for personalized medications. Charles W. Hull first proposed 3D printing technology in 1986, also known as 3D rapid prototyping or additive manufacturing technology–object technology [3]. Since then, with the increasing applications of digital technology and the development of the manufacturing industry, 3D printing technology has made significant progress and has been successfully applied in many fields of oral medicine, such as maxillofacial surgery, restoration, orthodontics, and tooth root canal preparation. Compared with other fabrication methods, 3D printing technology is more compatible, easy to produce, and cost-effective. Additionally, it provides the versatility of printing multiple biological materials and structural objects with complex designs alone or in combination. 3D printing, also known as rapid prototyping technology, is a modern technology that enables the construction of 3D objects from computer-aided design digital models [4]. This technology can be used to develop drug delivery systems where porosity is essential to achieving acceptable levels of biocompatibility and biodegradability and improving therapeutic efficacy. 3D printing allows users to control the dosage of each ingredient to perform a specific purpose and improve the formulation of drug delivery systems. This technology makes it feasible to manufacture drug products by combining multiple nanodrugs into different parts and releasing them from those parts at a predetermined rate. Furthermore, this 3D printing technology can potentially translate personalized treatments to other age groups through design flexibility and precise dosing. In recent years, the potential use of this technology has been realized in clinical situations where patients will receive personalized medicine according to their needs. 3D printing technology can accelerate drug research and development and achieve efficient production through an all-round data drive. The highly digital nature of this technology suggests that it can be replicated and deployed across geographies to address product supply challenges. Therefore, once 3D printing, a novel and more advanced type of pharmaceutical technology, achieves breakthroughs in product development and regulations and is applied in good manufacturing practice (GMP) drug production, more and more people in the industry will quickly realize its advantages and convenience [5].

This review discusses a comprehensive overview of how tailoring selected parameters (i.e., accurately selecting appropriate printing methods, materials, and printing parameters based on expected applications and behaviors) can better facilitate the development of optimized 3D printed products and how dynamic 3D printing strategies (designed to adapt to the needs of the user) can be deployed for printing materials that vary with time or stimulation to overcome many of the inherent limitations of traditional 3D printing technology. Furthermore, this review discusses the mutually beneficial collaboration of nanotechnology and additive manufacturing in the development of nano drug delivery systems. Moreover, it emphasizes applications, difficulties, and potential future developments while concentrating on the revolutionary potential of 3D printing at the nanoscale, provides a comprehensive insight into the critical prospects of 3D printing in the future and the essential requirements of 3D printing, such as the ability to program materials, print on multiple materials, and use exact nano design for fine conversion and even medical uses.

## 2 3D printing technologies for pharmaceuticals

**Fig. 1:**
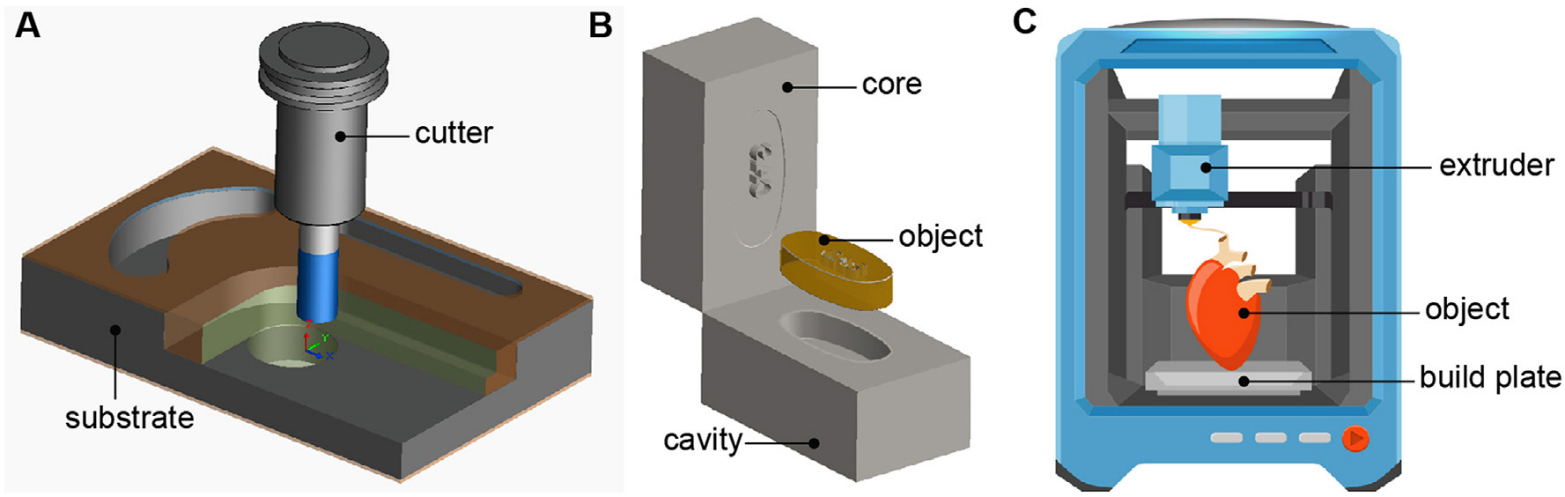
Illustration of three types of manufacturing methods: additive, formative, and subtractive. (A) In subtractive manufacturing, material-removing machines treat a block of material with a computer design to create the final three-dimensional (3D) object and a significant amount of residual material. (B) Formative procedures begin with the bulk material and shape it into its ultimate form, such as through injection molding or shaping. (C) In additive manufacturing, a starting material is processed by a 3D-printing device, which deposits the exact amount of material required layer by layer until the final 3D object is made.

As with traditional pharmaceutical methods, excipients are essential in developing and producing 3D-printed drugs. Moreover, it is critical to define the critical quality attributes of the drug substance early in drug development by understanding the properties of the active pharmaceutical molecule (API) [6,7]. The development history of manufacturing has roughly gone through three stages: The first stage is “subtractive manufacturing,” where the required parts are obtained by removing unnecessary parts of the material, such as logging, hand carving, and lathe cutting. The second stage is “equal material manufacturing”; the required products are obtained through molds and other methods, while the material quality remains unchanged. In the current pharmaceutical industry, the tableting technology of solid preparations is equal to material manufacturing. The third stage is “additive manufacturing,” called 3D printing technology. This is not to say that 3D printing can increase the number of materials, but that it can integrate computer-aided design, material processing, and molding technology based on digital blueprints through software and computer numerical control systems to “accumulate layer by layer” of materials, adding up bit by bit (Fig. 1). The method of manufacturing products gives 3D printing unparalleled advantages in precision manufacturing; it does not require molds and is more accessible in design; and it is significantly ahead of traditional manufacturing technology in the “four modernizations” of technological innovation, automation, digitalization, and intelligence [[8], [9], [10]].

Traditional pharmaceutical technology uses hot melt extrusion to prepare API into amorphous solid dispersions (ASD), grind it to obtain granules or powders of specific particle sizes, mix them with other formulation ingredients (such as binders, disintegrants, lubricants), and assemble them into tablets or fill them into capsules [11]. The final dosage form can be produced immediately without the requirement for grinding, granulating, and tableting processes by using a 3D printing method based on the extrusion principle. The above process can obtain optimal drug release kinetics by designing the tablet structure and solving the pulse release problem, which is difficult to achieve with traditional tablets. In addition, the formulation composition of 3D-printed pharmaceutical preparations is usually more straightforward because the 3D printing process differs from the traditional tableting process and does not require various additional excipients to achieve successful tableting. Therefore, there will be fewer raw material compatibility issues in 3D-printed pharmaceuticals, and 3D-printed tablets usually exhibit favorable long-term stability.

**Fig. 2:**
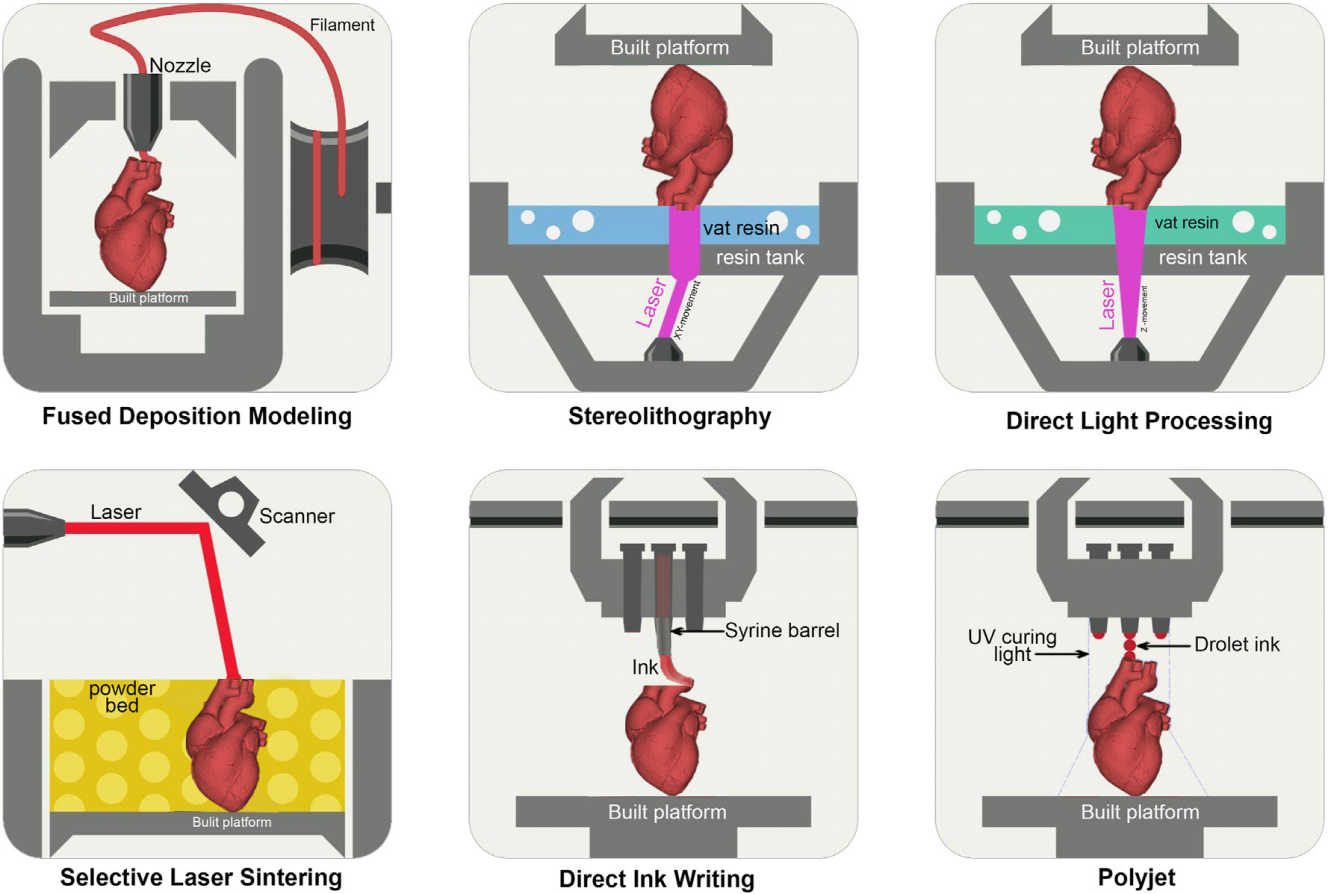
A schematic overview of the most commonly utilized 3D printing techniques: fused deposition modeling, stereolithography, digital light processing, selective laser sintering, direct ink writing, and material jetting (Polyjet). Copyright permission from Ref. [14].

3D printing technologies applied and researched in the pharmaceutical industry are divided into three categories: powder-based molding technology, liquid-based molding technology, and material extrusion-based molding technology. Powder-based molding technologies include powder drop technology (Drop on Powder, also known as powder bonding technology) and selective laser sintering technology (SLS). Liquid-based molding technologies include drop-on-drop and stereolithography (SLA) [[12], [13], [14]]. The fused deposition modeling technology, the pressure-assisted semi-solid technology, and Triassic's first hot melt extrusion deposition technology (MED) are all molding methods that use material extrusion (Fig. 2). Aprecia's Spritam is produced using powder bonding technology and has been successfully approved for marketing. Therefore, powder-based molding technology has always been the focus of the industry and has significantly progressed to achieve large-scale production. Aprecia and other pharmaceutical companies are working on applying multi-particle technology and nanotechnology to 3D-printed pharmaceuticals [[15], [16], [17], [18]]. SLS technology uses a laser system to fuse powder particles to achieve 3D structures. Unlike the traditional spray-drying formulation process, SLS does not require solvents or the spraying of any liquid; therefore, it has gained widespread attention. In recent years, molding technology based on material extrusion, especially technology related to hot melt extrusion (HME), has shown significant advantages in preparing ASD and has begun to garner the attention of the industry. Among them are the most popular advanced melt injection molding technology and hot MED technology [19,20].

### 2.1 Significance of nano drugs

The combination of 3D printing technology and nanomedicines is a revolutionary development in the pharmaceutical sector. Because of their minuscule particle sizes, nanomedicines provide more accurate and effective drug delivery. Pharmaceutical companies can design complex, customized medication delivery systems by combining 3D printing technology with these tiny compositions. Customized medication formulations and structures can be created using 3D printing, enabling targeted therapeutics and controlled release mechanisms. This combination makes it possible to produce drugs that are unique to each patient, allowing for individualized treatment plans. The integration between nanomedicines and 3D printing, which improves therapeutic outcomes and streamlines the production process, demonstrates the potential for a new era in personalized medicine. Nanotechnology is a scientific and engineering discipline that focuses on the deliberate design, fabrication, and utilization of structures, devices, and systems by precisely manipulating nanoscale atoms and molecules. The nanoscale is defined as having dimensions of 100 nm or less, equivalent to one hundred millionth of a millimeter. Due to its rapid development and combination with other high-tech and novel technologies, people need to understand the concept of nanometers [21]. In response to the hype regarding nanometers in our current society, a nanometer is a length measurement unit like a meter [22]. If it is called a micromillimeter, the concept it expresses will be the same. People have observed that when a substance is small to a certain extent, its physical and chemical properties will change, such as in conductivity and molecular structure. In nanomedicine, nanoscale substances are not used; rather, the dispersion property of medicine is. With scientific advancements, when drug particles are developed at the micron level, people naturally think about whether they can be made smaller. Therefore, nanotechnology is applied to the field of medicine. Because of the small size of nanomedicine and its favorable dispersion, it can be more easily absorbed by cells, thereby improving the bioavailability of the drug. Hence, people began studying nanoparticles and drug formulations. The production of nanomedicine is more complex than crushing the drug into particles with a size of 1–1000 nm. Each nanomedicine is composed of a carrier and a central active ingredient. Zhou Wenzhong et al. [23] made a vivid metaphor for a nanoscale dumpling. The drug is the filling, and the carrier is the skin. As the dumpling filling, the drug is the active ingredient that attacks disease cells, while the carrier, as the dumpling skin, plays the leading role. This analogy attempts to demonstrate the role of transportation and protection (such as targeted drug release, controlled drug release, sustained drug release, prolonged release, and pH maintenance). As the carrier and active ingredients are refined to the nanometer level, which is approximately the same size as the cell organelles, they can be better absorbed and utilized. On this basis, researchers can write many articles on the carrier to make it meet people's requirements. This can be done by adding a surface modification layer to the carrier and using interaction principles like the antigen-antibody reaction and the attraction of positive and negative charges. This way, the drug can find the target cells and treat them specifically instead of treating all cells, even if they are healthy, as regular drugs do. This surface modification layer is like the navigation device of a guided missile, allowing the drug to be concentrated in the target cells. Then, with the same amount of drug application, the concentration of the drug inside the target cells will be higher, especially during the treatment of cancer cells. Since drugs that kill cancer cells are harmful to normal cells, this kind of pertinence and purpose are essential. Another application of modified carriers is the slow and controlled release of drugs. Many pathogens cannot be killed instantly but require the sustained action of drugs. If the drug can be released slowly and continuously to keep the concentration in the cells stable, the pathogen can be killed at a relatively low dose. At the same time, drug resistance will not be caused by an instantaneous overdose. A suitable carrier is needed to release the active ingredients wrapped inside bit by bit, like a macroscopic capsule. The drug concentration curve within the cells will have a trapezoidal configuration, as opposed to the previous zigzag pattern. This particular form of nanomedicine is anticipated to exhibit a profound therapeutic impact, minimal adverse reactions, and a notable rate of drug absorption.

### 2.2 Drug delivery mechanism and design with nanotechnology

Drug delivery refers to the methods, preparations, storage systems, or related production technologies that deliver drug compounds to the target site or organ of the human body to achieve the desired therapeutic effect [24]. Drug delivery aims to change the pharmacokinetics and drug specificity of the drug by forming a formulation with different excipients, drug carriers, and drug devices [[25], [26], [27]] and significantly improve the bioavailability and duration of action of the drug in the body to improve the therapeutic effect of the drug [28,29].

#### 2.2.1 Guiding principles for controlled drug release

**Fig. 3:**
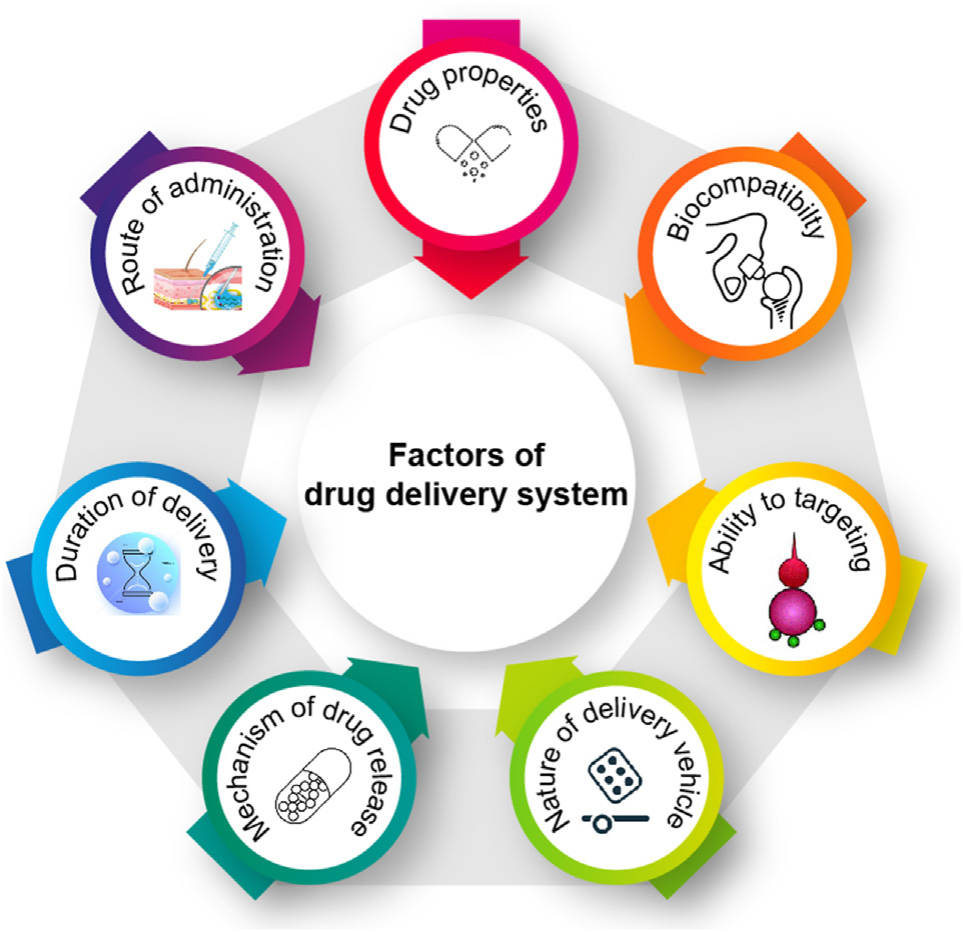
Requirements for designing drug delivery systems.

Drug delivery methods are intended to get therapeutic compounds into the body. They range from contemporary methods to traditional ones, like chewing medicinal plants. Due to the inconsistencies and homogeneity of the early methods, a variety of delivery systems, including pills, syrups, and capsules, were developed in the eighteenth and nineteenth centuries. A lot of people use plant extracts; the way medicinal agents interact with particular cell types typically determines how effective they are. Controlled drug delivery systems address this by taking into account factors like biocompatibility, targeting ability, release mechanism, administration route, delivery vehicle, and drug characteristics. As Fig. 3 summarizes, several independent variables make it difficult to distribute drugs precisely. Because it minimizes unpredictability and emphasizes the need for reliable, controlled systems, reliability is essential. The following section describes several methods of administering restricted drugs.

**Fig. 4:**
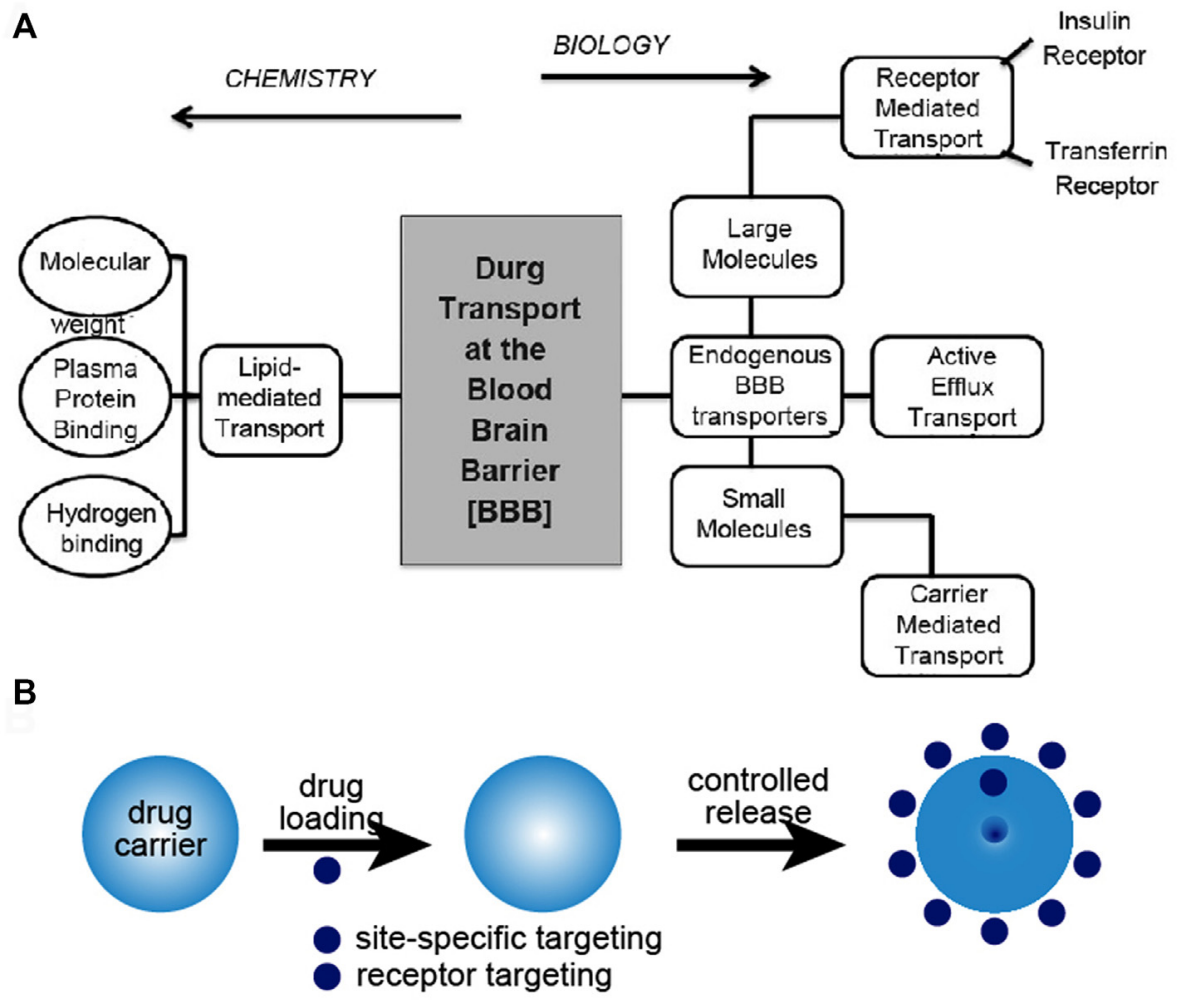
(A) Explanation diagram of the outline of a program for developing blood-brain drug targeting techniques based on both chemistry and biology, Copyright permission from Ref. [27]. (B) Schematic of the mechanism of a controlled drug delivery system.

Drug delivery is a concept highly integrated with formulation and route of administration, where route of administration is often considered a part of drug delivery research [30] (Fig. 4). Since the first controlled-release formulation was approved in the 1950s, although the number of novel drug developments has shown a downward trend, research on novel delivery systems has continued to make progress [31,32]. The application of nanotechnology in drug delivery is just one of its uses. Through nanotechnology, nanoparticles can carry drug molecules and deliver drugs to specific target areas in the body. Nanotechnology has several advantages for drug delivery, including precise, targeted delivery to specific cells, enhanced drug efficacy, and reduced toxicity to targeted cells. Nanoparticles can carry and deliver vaccines to cells that are difficult to reach with traditional delivery methods. However, there are still a few technical difficulties in using nanoparticles for drug delivery [33].

#### 2.2.2 The role of nanotechnology in drug delivery

Nanotechnology, which focuses on materials with sizes between 5 and 200 nm, is essential to the field of medicine, especially when it comes to the administration of drugs for different types of illnesses. Biodegradable nanoparticles contain drugs, protecting them from deterioration. By focusing on the areas that are impacted, this accuracy enables precise dosing that is site-specific and reduces side effects. Proteins, polysaccharides, and synthetic polymers are among the many materials that are used in nanoparticles; these materials are selected according to several criteria, including size, drug qualities, surface features, and delivery method. Solid lipid nanoparticles exhibit promise in antiviral and anticancer treatments, while gold nanoparticles are widely used in cancer therapy. An additional factor in customized medication administration is the use of nanofibers and nanosuspensions [34].

#### 2.2.3 Targeting mechanisms for nanoparticles

Two methods are available by which drug surfaces can bind nanoparticles and then load them into sick bodily tissues. Passive Targeting: Tumor tissues that are the intended target of the nanoparticles are more likely to receive them if they satisfy certain requirements, like continuing bloodstream circulation. Nanoparticles can easily get stuck in tumor tissues because of the unique properties of tumor cells, like their higher permeability and retention. This makes it easier to target the tumor with medication precisely. Tumor vascular dilatation with pores and gap junctions makes this possible by enabling selective accumulation in sick tissues. Active targeting and limitations on the specificity of nanoparticles are inherent to passive targeting techniques. If adding a targeted ligand to polymer-drug conjugates can help overcome these limitations, this is being investigated. Clinical trials encountered difficulties with direct antibody-drug conjugation, which limited its usefulness in treating cancer. Appreciating recent progress in liposomes, polymers, nanotubes, and metal-based drug carriers, it is now easier to attach more drugs to targeted nanoparticles and make their transport more effective. Preparing ternary-structure nanoparticles for more efficient delivery systems requires careful consideration of several issues [35].

Drug delivery systems that use 3D printing have difficulties when moving from laboratory testing to clinical trials, according to the majority of investigations, which have been conducted in vitro and in vivo in vertebrates. Meanwhile, Liang et al. effectively utilized a fused deposition modeling printer to fabricate a tunable mouth guard, including an adjustable pace of medication release. Table 1 provides examples of several 3D-printed drug delivery applications in different drug delivery systems (DDS).

**Table 1:** Diverse drug delivery systems in three-dimensional nanotechnology, along with descriptions and examples for specific diseases [36].

DDS	Explanation	Illustrative disease example
**Three-dimensional (3D)-printed drugs**	Drugs are created in a specific shape or structure by 3D printing. Drugs with better stability or biocompatibility can be developed thanks to this approach, which improves drug delivery to specific body regions.	Parkinson's disease, cystic fibrosis, and cancer
**Nanoparticles**	Tiny particles that are intended to deliver medications to specific bodily sites. These particles could be made of metals, lipids, or polymers, among other substances.	Diabetes, rheumatoid arthritis, and cancer
**Nanocarriers**	Medicines are delivered to specific cells using specially designed nanoparticles. These nanoparticles can be made to accumulate in particular organs or targeted to bind to particular cell surface receptors.	HIV/AIDS, cancer, and Alzheimer's disease
**Nanorobots**	Nanomachines are made to deliver drugs to specific bodily parts. These gadgets can be designed to go around the body and release medication in specific places.	Cardiovascular disease, stroke, and cancers

Delivery mechanisms and 3D printing have a revolutionary link that is changing conventional manufacturing and distribution procedures. Additive manufacturing, commonly referred to as 3D printing, makes it possible to produce complex, personalized goods on demand. Because it allows for decentralized production, this invention has a direct impact on the delivery chain. Products can be printed closer to the site of consumption rather than relying on centralized factories, which lowers transportation costs and has less environmental impact. This change makes just-in-time production possible, reducing the requirement for inventory storage and optimizing supply chains. The combination of delivery mechanisms and 3D printing thus signals a paradigm shift toward production systems that are more responsive, sustainable, and agile.

**Fig. 5:**
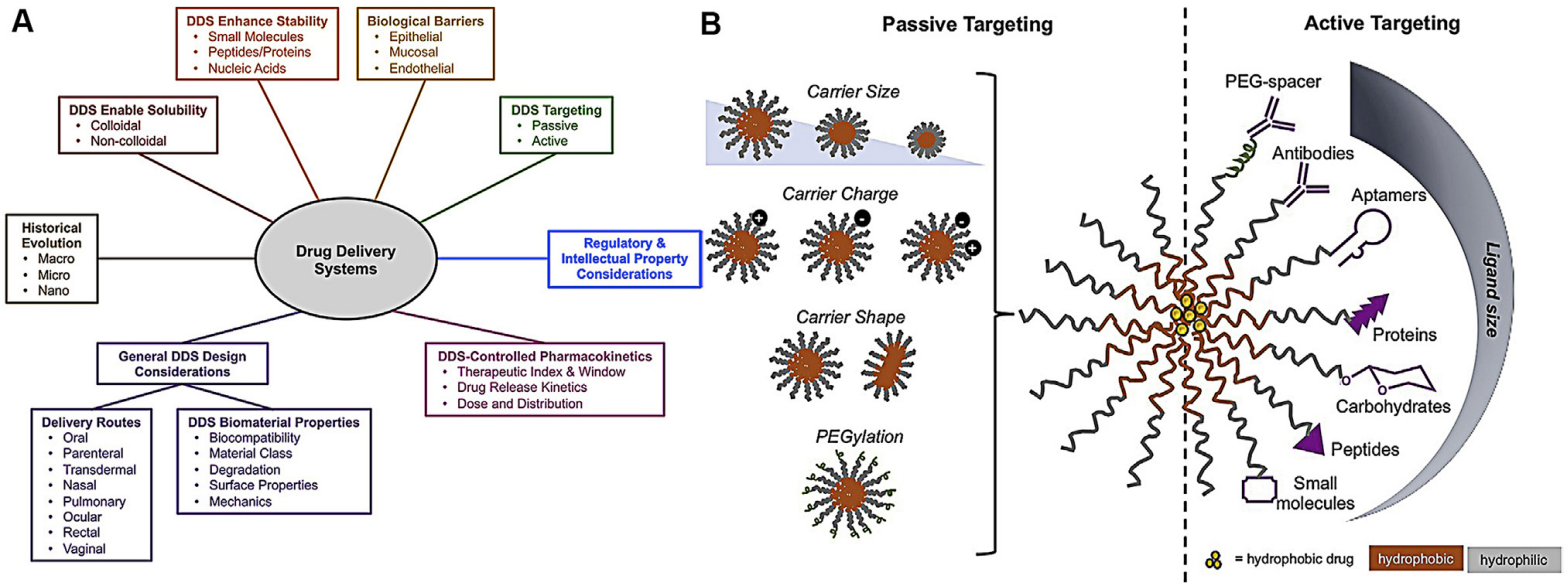
(A) Overview of the development of drug delivery systems (DDS) and (B) diagrammatic illustration of active and passive targeting of DDS. The physicochemical characteristics of the carrier, such as its size, charge, and shape, are essential for passive targeting. Furthermore, surface modification with poly (ethylene glycol) improves targeting and circulation time. The many ligands and their corresponding range of sizes are displayed in active targeting. Copyright permission from Ref. [37].

The third generation of drug delivery (Fig. 5) is concentrated on adjusting DDS and learning more about their behavior in vivo to expedite license development. The combination of AM with nanoscale drug delivery systems is revolutionizing the field of pharmaceutical development. With advantages including targeted therapy, improved bioavailability, and controlled release, nanotechnology makes precise drug delivery possible. Personalized medication delivery systems that take into account various characteristics, such as anatomy and treatment requirements, can be created more easily due to additive manufacturing, especially 3D printing. By providing better medication stability and controlled release, nanoscale-engineered nanoparticles maximize therapeutic efficacy while decreasing off-target effects. Multiple drugs can be encapsulated in a single nanoparticle to assist combination therapy. AM techniques, such as 3D printing, enable the customization of complex medicine formulations for unique patients. Embedding nanoparticles in 3D-printed scaffolds for tissue engineering and regulated drug release is one way to integrate nanoscale drug delivery with AM. Sensitive drug release systems adjust to changes in their surroundings by utilizing stimulus-responsive nanoparticles. Biosensor integration allows for real-time monitoring and supports adaptive medication release, while 3D-printed microfluidic devices provide accurate drug flow control [37].

The structure of 3D printing involves material science, but hardware, software, and different design mechanisms are necessary. 3D printing using metal and carbon fibers is now prevalent. Moreover, it has become affordable and available to everyone, whereas initially, it was limited to industrial companies with more financial resources and greater research capabilities. Graphene, sometimes referred to as a “semi-metal,” is touted as a material full of magical abilities due to its ultra-lightweight and incredible strength—far more than diamond or even steel. George et al. [38] explored the application of graphene in 3D printing and the possible toxicity of exposure to cells. Graphene is unique in that it is a two-dimensional structure with incredible strength, rigidity, and noteworthy qualities such as electrical conductivity. Concerns exist regarding its safety when used in biomedical applications, such as creating scaffolds to promote living cell growth or for drug delivery, bioimaging, and even biosensing. Integrating drug-releasing polymer layers onto sea-faring microcarriers is among the most promising treatment strategies for a variety of diseases. Bernasconi et al. [39] describe the fabrication of 3D-printed and wet-metalized microdevices for targeted drug delivery. The microtransmitters use stereolithography and are coated with a range of materials to give them specific functionality, such as magnetizability and chemical inertness. Polypyrrole has been electrodeposited as the top layer in both bulk and nanostructured forms to add drug delivery properties. The fabricated microdevices are characterized from a morphological and functional perspective.

**Fig. 6:**
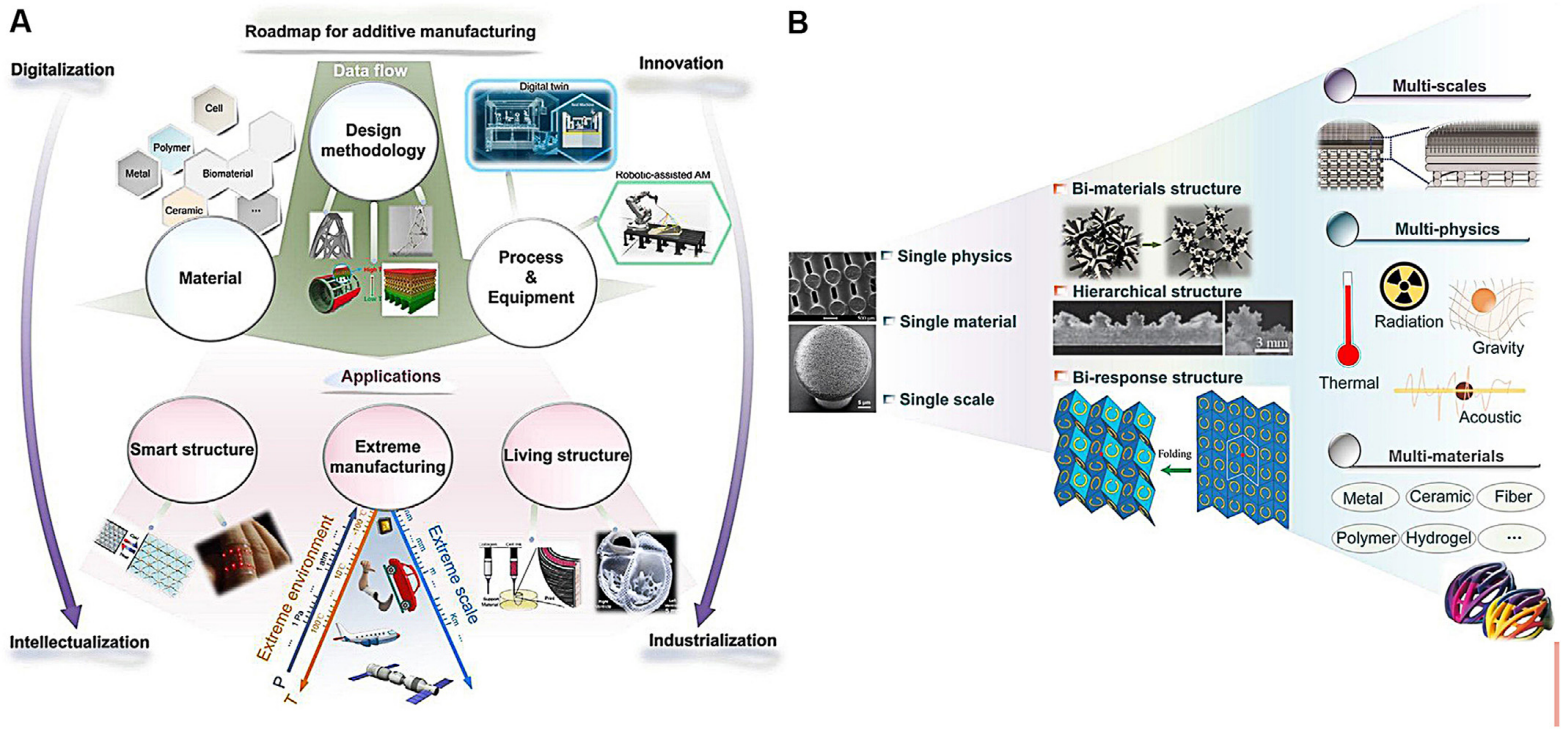
(A) A schematic representation of the additive manufacturing roadmap. (B) Many specific mechanisms are available to be solved, even with the quick advancement of additive manufacturing technologies for building intelligent structures. Copyright permission from Ref. [40].

Drug delivery refers to the methods, preparations, storage systems, or related production technologies that deliver drug compounds to the target site or target organ of the human body to achieve the desired therapeutic effect. The goal of drug delivery is to change the pharmacokinetics and drug specificity of the drug by mixing different excipients, drug carriers, and drug devices to make a formulation. This greatly increases the bioavailability and length of action of the drug in the body, which makes the drug more effective (Fig. 6). Drug delivery is a concept that is highly integrated with formulation and route of administration, where route of administration is often considered a part of drug delivery research. The increasing prevalence of chronic and infectious diseases and a greater understanding of drug pharmacology, pharmacokinetics, and pharmacodynamics make the study of drug delivery systems increasingly crucial in the field of drug development. Now, AM systems are trying to combine various physical fields and offer dynamically responsive n-dimensional printing for complicated multi-scale structures using sensing and actuation tools that work well together [40].

#### 2.2.4 Impact of 3D shape and structure on controlled drug delivery using nano-carriers

**Fig. 7:**
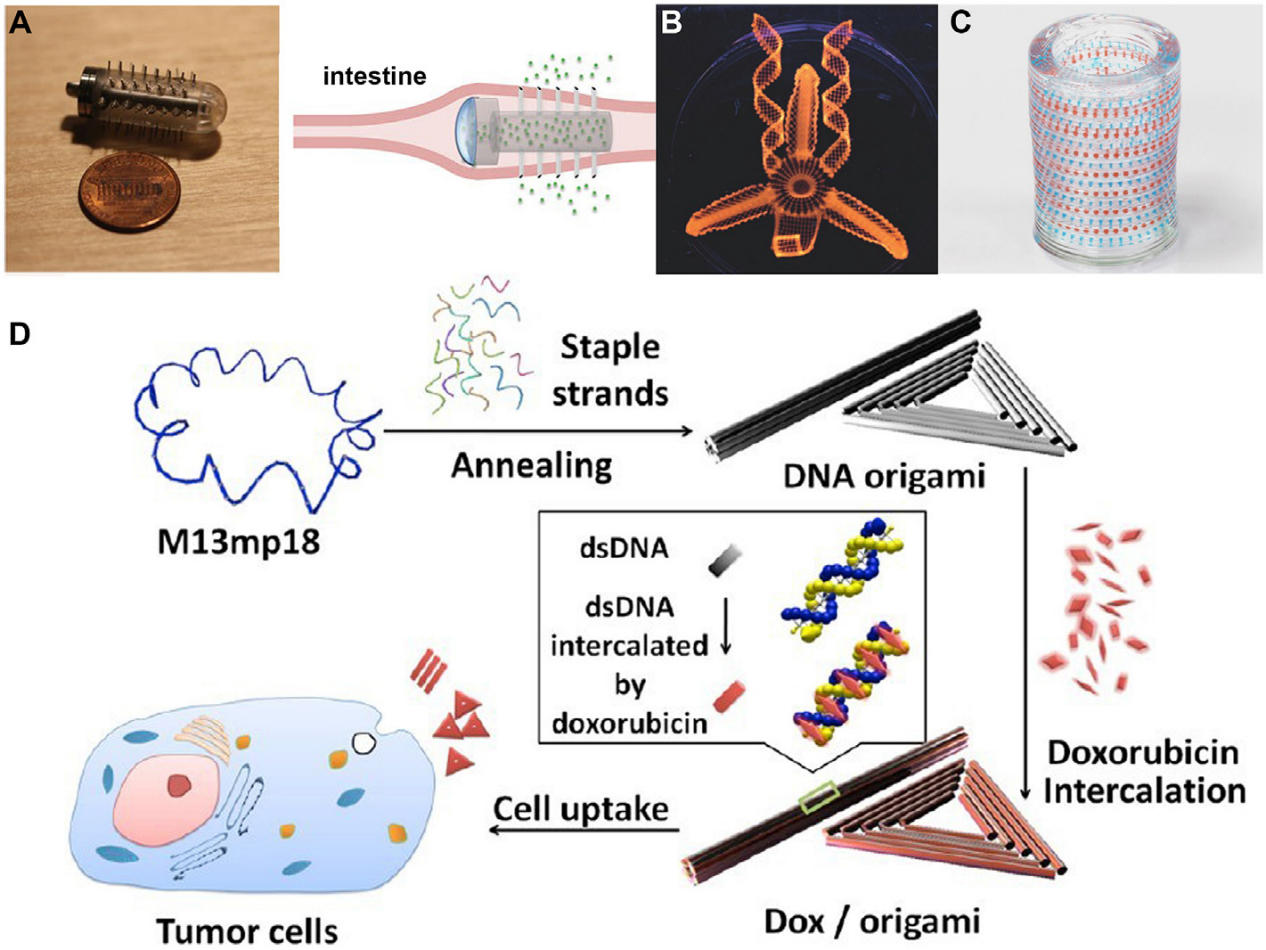
(A) Delivery of specific insulin in the gastrointestinal tract is made easier by microneedle-coated capsules. A schematic depicting the retention of the device in the gastrointestinal tract for continuous insulin release is shown on the right, while an optical image on the left shows the full device. Copyright permission from Ref. [41]. (B) A three-dimensional (3D)-printed hydrogel flower that is loaded with PLA and poly (lactic-*co*-glycolic) acid particles coated in an Au nanorod. Copyright permission from Ref. [42]. (C) 3D multiplexed capsule arrays directly printed in square and cylindrical hydrogel matrices are shown in optical images. Copyright permission from Ref. [43]. (D) A diagram showing how to make DNA origami triangles that target tumor cells with an anticancer medication. The blue-colored strands of M13mp18 phage genomic DNA are folded into rod- and triangle-shaped nanoparticles. These particles are carried into tumor cells after being intercalated with doxorubicin, which is indicated in red. Copyright permission from Ref. [44].

Growing interest exists in the effects of particle shapes on medicine delivery efficiency. Changes in particle shape affect functioning, yet the exact function of 3D shape remains unclear based on experimental findings. The main benefit is that the drug particle specificity can be improved to target particular organs. By developing a microneedle-coated capsule, Traverso et al. [41] solved the difficulties associated with delivering sensitive biologics by conventional oral ingestion by targeting the gastrointestinal system. This method enhanced the ability of the gastrointestinal tract to receive continuous insulin infusions without experiencing negative side effects (Fig. 7A). The 3D-printed hydrogel-based microfish hold great potential as targeted drug delivery vehicles, as evidenced by their capacity to swim within the body for up to 2 h. Either iron oxide for magnetically directed propulsion or platinum nanoparticles for chemical propulsion are used to accomplish this. The minute details demonstrate how complex drug particles may be produced via 3D printing. More intricate 3D-printed hydrogel designs are an example of recent developments, as shown in Fig. 7B [42]. With this technique, users can precisely control the release of the medication payload by varying the nanorod length. Furthermore, as shown in Fig. 7C [43], the particles can be formed into complex arrays and encased in a hydrogel apparatus. Jiang et al. [44] reported that 3D nanostructures of doxorubicin-containing DNA complexes were used to treat breast cancer. The method for creating an origami triangle DNA target for cancer cells that incorporates doxorubicin is shown in Fig. 7D. Tests for biodistribution showed a considerable buildup at the locations of the tumors, which peaked 6 h after injection and persisted for 24 h. After 24 h, triangle accumulation signals were twice as high as those observed in DNA tubes and squares. Organ explants exhibited a significant accumulation of triangles at tumor locations, which set them apart from the tubes and squares that are common in kidneys and livers. This phenomenon fits with traditional drug-using trends. 3D DNA origami has the potential to be used in novel medication delivery systems.

#### 2.2.5 Polymeric formulation

During the latter part of the 1980s, several 3D-printing technologies surfaced, akin to the proliferation of mushrooms following rainfall. Therics, a pioneering pharmaceutical business, was founded in 1996 with the ambitious goal of integrating 3D-printing technology into the conventional pharmaceutical industry [45]. In 2015, 3D-printed medicine became a reality. The FDA approved Aprecia's anti-epileptic drug, Spritam. The drug uses 3D-printing technology to have an internal porous structure that can quickly disintegrate and solve the clinical needs of dysphagia. The release of the first 3D-printed medication in the world signifies the regulatory approval of this new technology for printing drugs. Additionally, it has set off a wave of research on 3D-printed drugs. More than 50 companies and institutions worldwide have successfully entered the field of drug 3D printing, including dozens of multinational pharmaceutical companies [46].

Nanomaterials refer to materials with at least one dimension in the nanoscale range (0.1–100 nm) in three-dimensional space or as basic units. Due to their small size, large specific surface area, high surface energy, and a large proportion of surface atoms, nanomaterials have five special effects different from those of macroscopic material systems: volume effect, surface effect, quantum size effect, macroscopic quantum tunneling effect, and dielectric confinement effect [[47], [48], [49], [50]]. Gold nanorods are a typical representative because of their strong absorption in the near-infrared light region of the plasmon absorption peak; as a result, they have good photothermal conversion efficiency and are often used in the field of biomedicine, such as for drug delivery and cancer treatment.

**Fig. 8:**
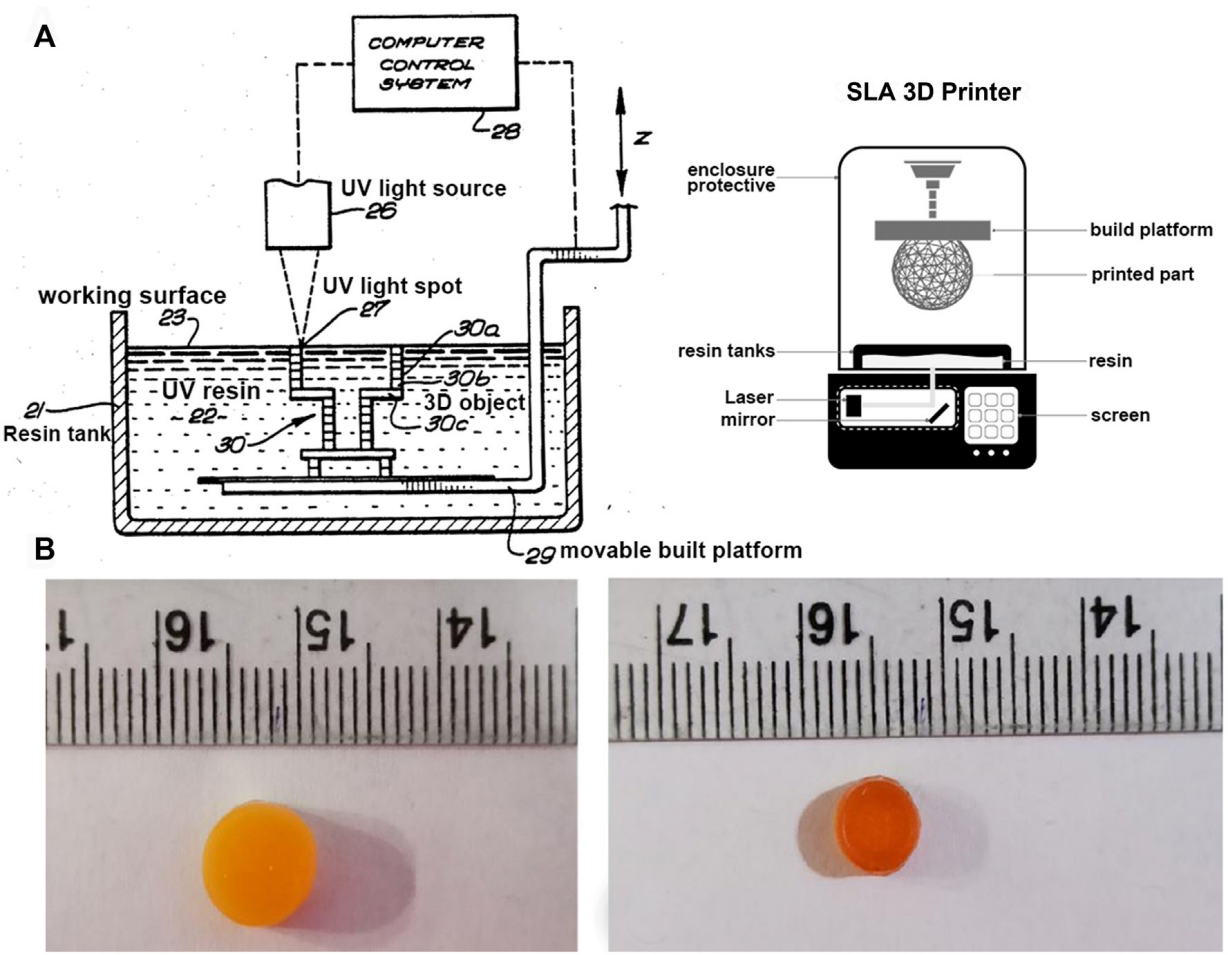
(A) An illustration introducing and showing the term and concept of stereolithography: (21) UV curable material container, (22) UV curable materials, (23) working surface of the fluid medium, (26) UV light source, (27) spot beam of UV light, (28) computer, (29) movable elevator platform inside container, (30) 3D object, and (30 ac) step-wise buildup-integrated laminae of a 3D object and SLA 3D printer graphical model. Copyright permission from Ref. [14] (B) Dimensions of an air-dried nanocomposite drug delivery device that has been SLA-printed. The diameter and height of a nanocomposite pill as printed and air-dried. Copyright permission from Ref. [52].

Manisha Pandey et al. [51] described recent advances and challenges faced by 3DP technology in developing oral drug delivery. Despite the advantages of 3DP technology in drug delivery systems, there are challenges in drug stability, safety, and clinical applicability. Nonetheless, 3DP has great potential for developing drug delivery devices for future personalized medicine. Sharma et al. [52] successfully prepared nanocomposite pellets (Fig. 8). After testing, the composite nano-drug delivery system printed in this experiment exhibited mechanical elasticity and was deemed suitable for commercial and practical applications. The excellent swelling properties of nanocomposites give them significant advantages because high swelling properties lead to more efficient penetration and release through the matrix. The lower drug loading of composite nano pellets is due to the tendency of BBR-NPs to settle slowly during the photopolymerization 3D printing process. SEM micrographs further confirmed that the BBR-NPs were encapsulated in the matrix of the composite nano pellets. The difference between the nanoparticle size measured using scanning electron microscopy and differential scanning calorimetry and between the average particle size measured using differential scanning calorimetry and electron microscopy is the swelling of BBR-NPs during the SLA printing process and melting behavior. The faster release of BBR at an acidic pH is consistent with the significantly higher degree of swelling under acidic conditions. Since drugs are released from composite nanomaterials based on swelling and diffusion processes, the release pattern can be tuned by modulating the swelling behavior. Because SLA 3D printers are limited by filling density, filling density can be used as a controllable parameter for drug release. Therefore, to compensate for this limitation, pills can be printed with different proportions of cross-linking agents (PEGDA and PEG) and by modifying the design of the pill, such as by introducing a porous structure that affects pill expansion. The information gathered indicated that berberine hydrogel nanoparticles were made. These particles had an average size of 95.05 ± 4.50 nm and had lower drug concentrations. Here, high-fidelity (consistent with computer-aided design) stereolithography-assisted monolithic fabrication was achieved, and photocrosslinking was identified by Fourier-transform infrared spectroscopy. Electron microscopy revealed that the hydrogel nanoparticles were encapsulated in the pills during stereolithography. The composite nano-pellets had a high swelling degree in an acidic environment, and the release rate of berberine was 50.39 ± 3.44% after 4 h.

The flow lines of the organic phase through the nozzle and along the two mixing elements were simulated. The colored water flow represents the speed of the water flow. Simulation experiments by Peer Erfle et al. [53] produced effective and cost-effective customized drugs that are gaining traction in the pharmaceutical industry. The characteristics of poorly soluble drugs limit their oral and parenteral administration. Lipid nanoparticles containing poorly soluble pharmaceuticals will become viable options to address the issue of poor solubility as they offer quicker rates of dissolution. However, producing these lipid nanoparticles requires a lot of resources. The entire process includes multiple steps, such as the preparation of nanoparticles and the conjugation of drug carriers to the nanoparticles. During the production of nanoparticles, it is essential to manage a narrow particle size distribution to achieve the required range of 70–200 nm. For this purpose, microfluidic systems offer a more optimized solution than batch mixing techniques. Andreas Dietzel [54] has developed a pioneering solution through research in microfluidics to prepare monodisperse drug carrier nanoparticles. They used Nanoscribe's two-photon polymerization 3D printing technology to produce a complete microfluidic chip. The chip uses a unique micro-nano hybrid device for coaxial lamination and stable nanoparticle generation. The entire centimeter-scale microfluidic chip consists of a main channel connected to the lateral channel, a nozzle for coaxial injection, a series of 3D mixing elements, and an inlet filter to reduce contamination. This complex chip design stands out for its miniaturization and extremely high surface quality (e.g., the main channel with an inner diameter of 200 μm and the inlet filter with a pore size of 15 μm). Stretched and folded micro-nano components that mix organic and aqueous phases have complex 3D structures. In the past, traditional 2.5 D micro-nano processing and mass production using micro-nano injection molding were unable to manufacture such microfluidic systems due to the difficulty of molding the bottom incised structure and the open cylindrical area [55].

**Fig. 9:**
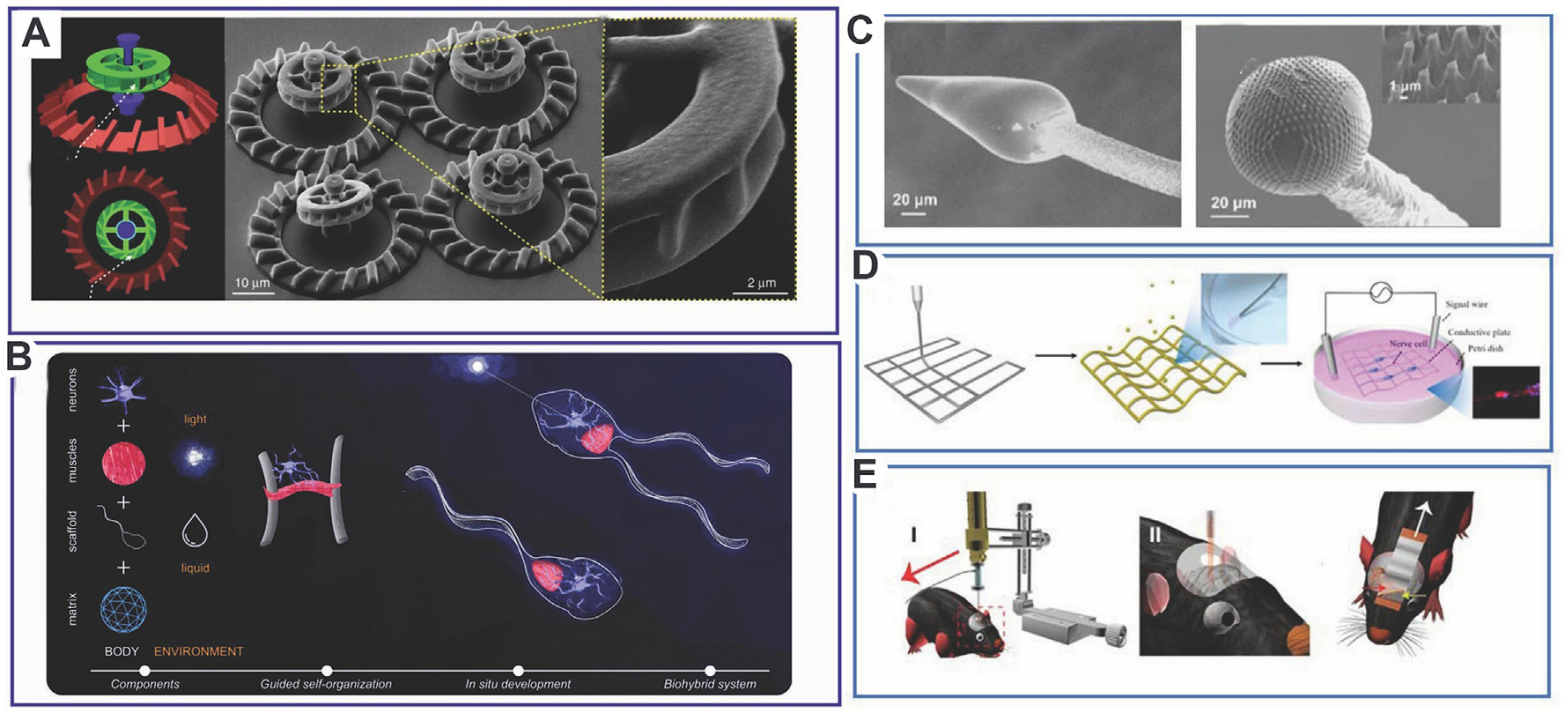
(A) Optical stimulation of genetically modified bacteria controls a 3D-printed biohybrid microsystem; the micromotor structure in 3D SEM photos of three-dimensional micromotors. (B) Schematic illustration of a biohybrid swimming microrobot driven by light stimuli. (C) Multiphoton lithography and pyrolysis are used to create neurointerface microelectrodes. (D) Diagram depicting the preparation of injectable Au-PCL scaffolds with excellent conductivity and a high-throughput nerve cell stimulation device. (E) 3D inkjet printing with carbon nanotube ink to connect the injected neurointerface to the recording equipment. Copyright permission from Ref. [57].

The “two-photon lithography” 3D printer that Nanoscribe, a German company that specializes in microscopic 3D printing, uses can print with micron or nanometer accuracy. In the medical field, nanorobots created by extracting proteins or cells from living organisms as printing raw materials are small and do not cause severe immune rejection in the living body. Therefore, they can be used in the human body for drug delivery, toxin cleansing, and targeted therapy. Treatments for glaucoma or diabetic macular edema are delivered via direct injection or eye drops. This method is effective but imprecise, and it often leaves the entire eye receiving medication. Therefore, scientists used nanoscale 3D printing to create spiral-shaped robots that are small enough to pass through the transparent jelly called vitreous, which is the primary building block of the eyeball. Relevant researchers added magnetic materials and a smoother layer to the robot and then pushed it into the eye to help the diseased area receive precise treatment. Researchers developed a 3D printing robot driven by bull sperm cells. Each biohybrid sperm microrobot is made from a combination of bull sperm and plastic 3D-printed microstructures covered with an iron-based coating (Fig. 9). Doxorubicin hydrochloride is a medication used to treat cervical cancer and can be placed on the head of the sperm. The metal coating allows the researchers to magnetically “guide” the robot to the precise location it needs to be. Afterward, the four movable arms on the microstructure release sperm cells that target cancer cells to treat cervical cancer patients [56].

Ultrasound-enhanced microchannel emulsification technology was used to look into a new way to make repaglinide nanoparticles that are enclosed in a polymer. Three different kinds of micromixers (T-type, Y-type, and F-type) were created utilizing SS-316 and the 3D printing concept. Next, serpentine microchannels were created. Parametric studies were performed on all three fabricated micromixers. The best results were obtained only for the Y microchannel in the microfluidic system, with a minimum particle size of 513.6 nm and an encapsulation efficiency (EE) of 75.4%. In a few microchannels, ultrasound was used to improve convective mixing between fluids that do not mix to reduce the size of the drug particles further and raise the EE percentage. Compared with the microfluidic system, the particle size and EE of the ultrasonic microfluidic system were significantly improved. Experimental results show that an ultrasound-enhanced microfluidic system achieved a minimum particle size of 75.4 ± 1.3 nm and 82.9 ± 0.2% EE [58].

The invention describes how to make and use a 3D bionic cell implant that can release microRNA nucleic acid drugs. Additionally, it provides a new way to administer biological nucleic acid drugs, makes it less likely that nucleic acid drugs will break down in living organisms, and provides a way to prepare 3D bionic implanted cells in living organisms. The invention inserts microRNA expression plasmids into engineered cells that make cellular exosomes. These exosomes contain nucleic acid molecules that can be released from cells. At the same time, the engineered cells are loaded with approved 3D-printed bionic implants. The genetically modified cells can release the recombinant nucleic acid drug miR377 in the form of exosomes in a stable and long-lasting way by mimicking the way nutrients move through blood vessels to nearby tissues. This makes it possible to create vascular bionic linear implants that can make drugs on their own inside the body. The current invention uses the 3D bionic cell implant miR377, which is resistant to esophageal cancer. This genetically engineered cell creates a drug-endocrine platform technology for recombinant nucleic acid drugs [59].

**Fig. 10:**
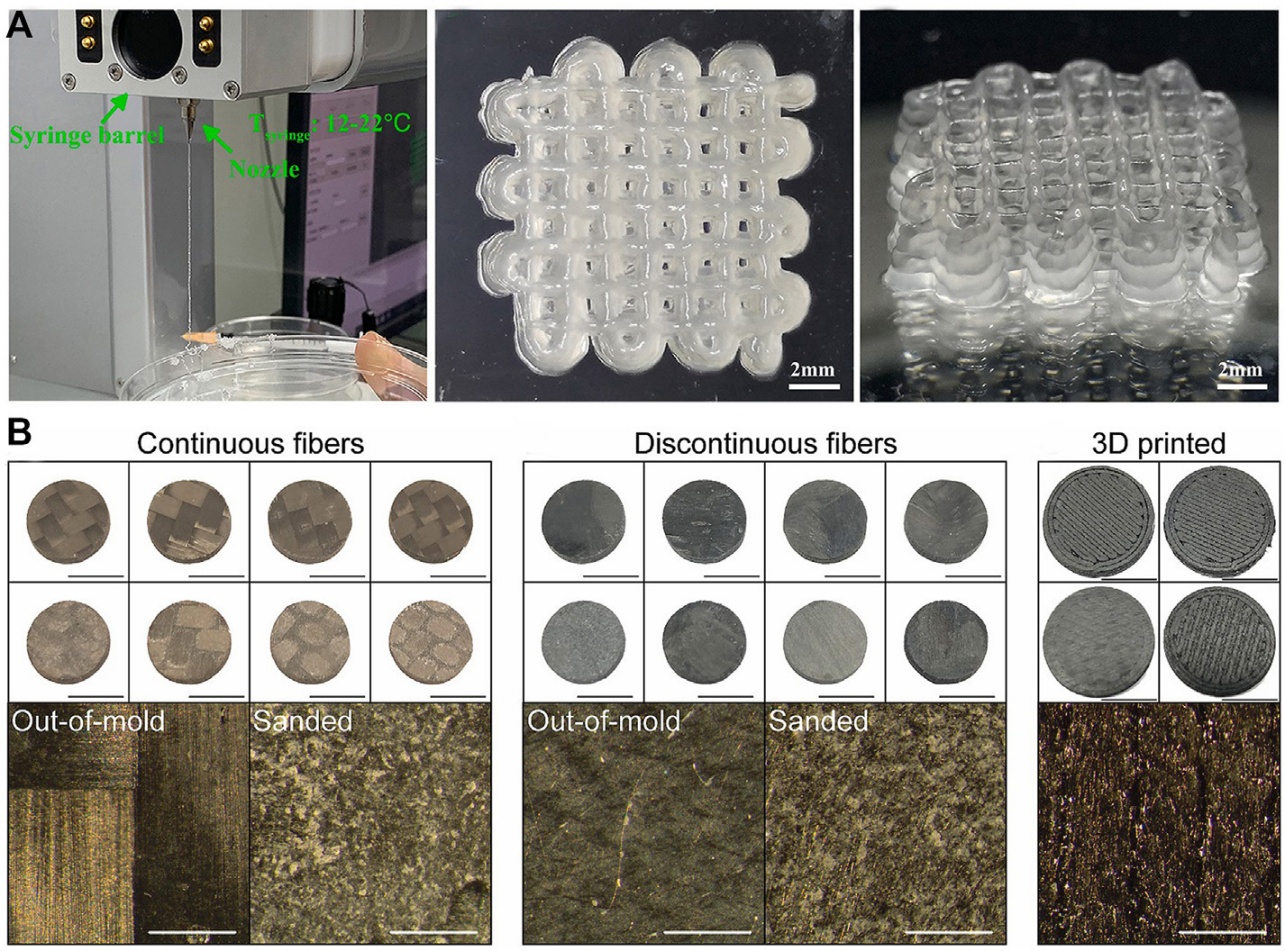
(A) T_syringe_ refers to the temperature of the syringe barrel in the 3D bioprinting process. Top and side views of the 3D-bioprinted scaffold. Copyright permission from Ref. [60]. (B) CFRP discs with continuous and discontinuous fibers were created using the Carbon Fiber-Sheet Molding Compound (CF-SMC). Out-of-mold samples are shown in the top row, from left to right: AMC-R3, EXP-R3, M81-R3, and M77-R3. Discs produced in 3D. CFRP and GFRP are in the top row, from left to right. KFRP and Onyx are in the bottom row, from left to right. Magnification of a representative 3D-printed disc in the bottom panel, and 5 mm is represented by the black scale bars. The white scale bars represent 500 μm. Copyright permission from Ref. [61].

Xin Sun et al. [60] used 3D bioprinting technology to prepare a novel scaffold that can promote bone repair, providing a novel idea and method for repairing such bone defects for the first time. They created a new bone repair scaffold that was made with 3D bioprinting and filled with bone marrow stem cells, bone morphogenetic protein 4 (BMP-4), macrophages, and a system for delivering factors (Fig. 10). Their scientific research and design first raised the critical issue of “preparing ink that can print such biological scaffolds.” The researchers innovatively proposed loading bone marrow mesenchymal stem cells, BMP-4, and macrophages into the bioink. At the same time, to shorten the bone formation time, a drug delivery system was added to the material: mesoporous silica nanoparticles, and inorganic nanoparticles used for drug delivery. This enabled them to use 3D bioprinting “bioink” to deliver living cells that promote bone growth and achieve better control of the distribution and deposition of osteoblasts. They progressed in selecting and regulating the standard ratio of the two types of macrophages. A formula that meets the requirements for 3D printing bone scaffolds with precise shapes and functional structures was successfully prepared. 3D printing relies on hardware, software, and design procedures, but materials science is at its foundation. Metal and carbon fiber 3D printing is standard; it is now more affordable and available to everyone, unlike when it was only available to industrial enterprises with more resources and research studies. Due to its ultra-lightweight and incredible strength—far greater than that of diamond or steel—graphene, sometimes called a “semi-metal,” is said to have “magical” properties [61].

## 3 Applications of 3D-printed nano drugs

In recent years, through continuous technological innovation and material research, 3D printing technology has been widely used in the pharmaceutical field in developing and producing small-molecule solid preparations to develop better pharmaceutical products and biological preparations.

### 3.1 Biomedical

In response to the development needs of novel technologies such as organ repair, 3D printing has become a model for the integration of biotechnology and material technology. However, at this stage, the bottleneck of 3D printing materials restricts the development of 3D printing technology. 3D printing of biomedical materials is challenging. Moreover, it is necessary to consider the strength, safety, biocompatibility, and degradability of tissue engineering materials. Currently, biomedical materials used for 3D printing mainly include metals, ceramics, polymers, and bioinks, characterized by a wide distribution range but few types. Medical metal materials currently mainly include materials used for biomedical printing, such as titanium alloys, cobalt-chromium alloys, stainless steel, and aluminum alloys. With the emergence and development of nano-3D printing technology, nano-powder printing materials have gradually become a research hotspot, and metal powders occupy an essential position in the 3D printing powder market. Medical inorganic non-metallic materials include bioceramics, bioglass, oxide, calcium phosphate, and medical carbon materials.

**Fig. 11:**
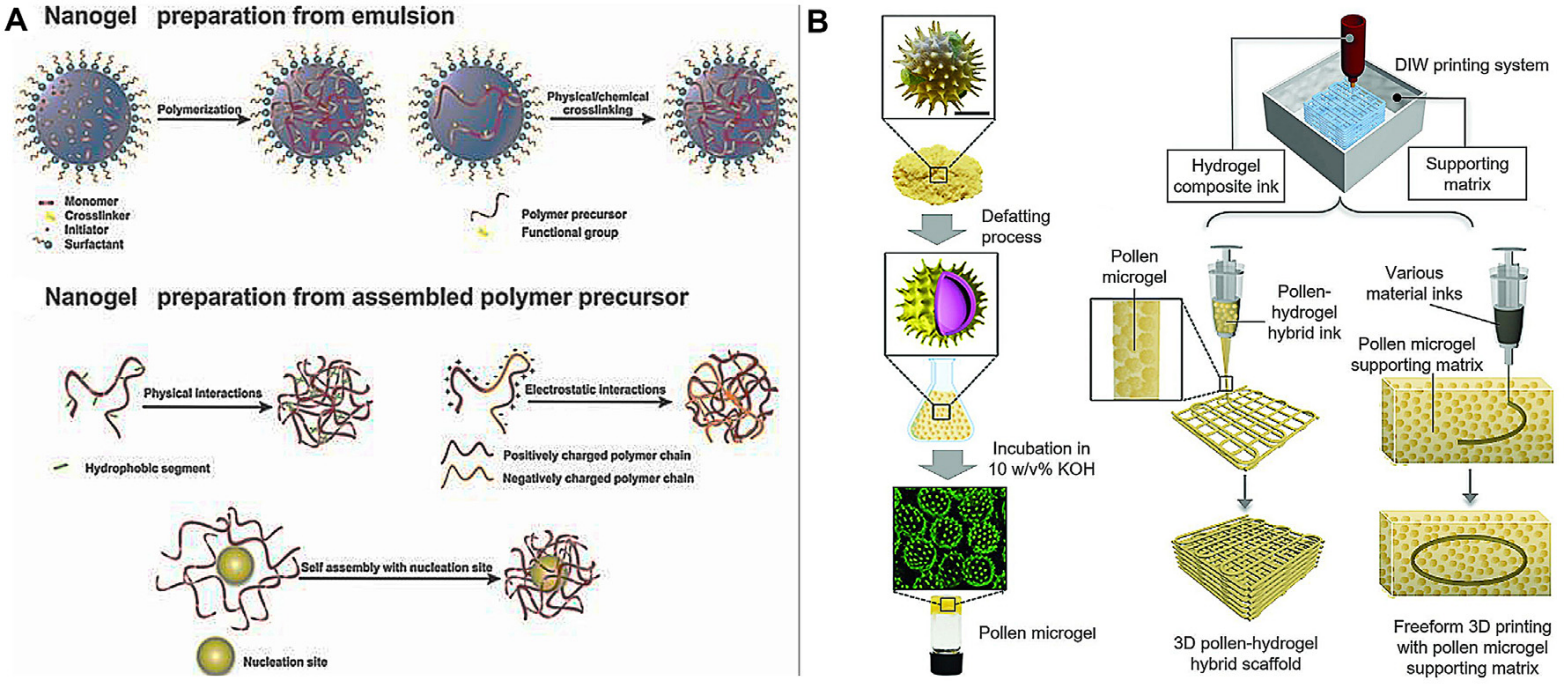
(A) Synthetic approaches for producing specific NGs are depicted schematically. Synthesis of NG from emulsions and preparation of NG from assembled polymer precursors. Copyright permission from Ref. [62]. (B) Pollen microgel illustration as a bioink and supporting matrix material for 3D printing applications. Microgel production from natural pollen grains. Incubation of pollen grains in an alkaline solution results in a microgel with controllable rheological properties. The scale bar represents 10 m. A DIW printing system with hydrogel-based inks and with or without a supporting matrix, where pollen microgels can be combined with hydrogel materials to form hybrid materials that can serve as biocompatible inks with new functionalities, such as controlled release, and a pollen microgel medium acting as a supporting matrix for freeform 3D printing with a variety of inks. Copyright permission from Ref. [63].

With the breakthrough progress of nanomedicine, innovative drug delivery systems have been rapidly developed, in which nanocarriers play a vital role in disease treatment. Compared with ordinary drugs (which have many limitations such as a short half-life, poor drug delivery, and non-specific systemic toxicity), stimulus-responsive nanogels (NGs) can deliver anti-tumor drugs to specific sites and improve therapeutic effects without needing specific stimulation. Abedi F et al. [62] demonstrated novel advances in stimulus-responsive NGs such as light-, temperature-, redox-, magnetic-, and pH-responsive NGs. The authors summarize the preparation, biomedical applications, current advances, and challenges of stimulus-responsive NGs to achieve effective cancer therapy (Fig. 11). Shengyang Chen et al. [63] have reported a way to use sunflower pollen to develop 3D-printing ink materials that could be used to create parts useful for tissue engineering, toxicity testing, and drug delivery. This pollen-derived ink can maintain its shape when deposited onto a surface, making it a viable alternative to inks currently used for 3D printing (also known as bioprinting) in the biomedical field. Inks are often soft and delicate, making it difficult to maintain the desired 3D shape and structure of the final product as the bioprinter deposits the ink layer by layer.

### 3.2 Tissue engineering

The invention discloses a method for preparing a skin tissue engineering scaffold based on 3D bioprinting technology and an in vitro cytotoxicity testing method for the scaffold, including the preparation of a high-strength cellulose nanofiber/gelatin composite hydrogel for printing the scaffold, the tissue engineering scaffold 3D printing process, and the scaffold cross-linking process. The present invention uses 3D bioprinting technology to solve the high porosity and precision requirements of tissue engineering scaffolds. As the filler of GEL, CNF improves the mechanical strength of GEL. The printed scaffold is soaked in a genipin solution for cross-linking. This method produces a skin tissue engineering scaffold with good mechanical properties, no toxic side effects, and no immune rejection reaction. At the same time, using 3D printing technology to prepare tissue engineering scaffolds has the advantages of convenience, speed, and easy control. The size and shape can be customized according to the depth of a patient's wound [64].

**Fig. 12:**
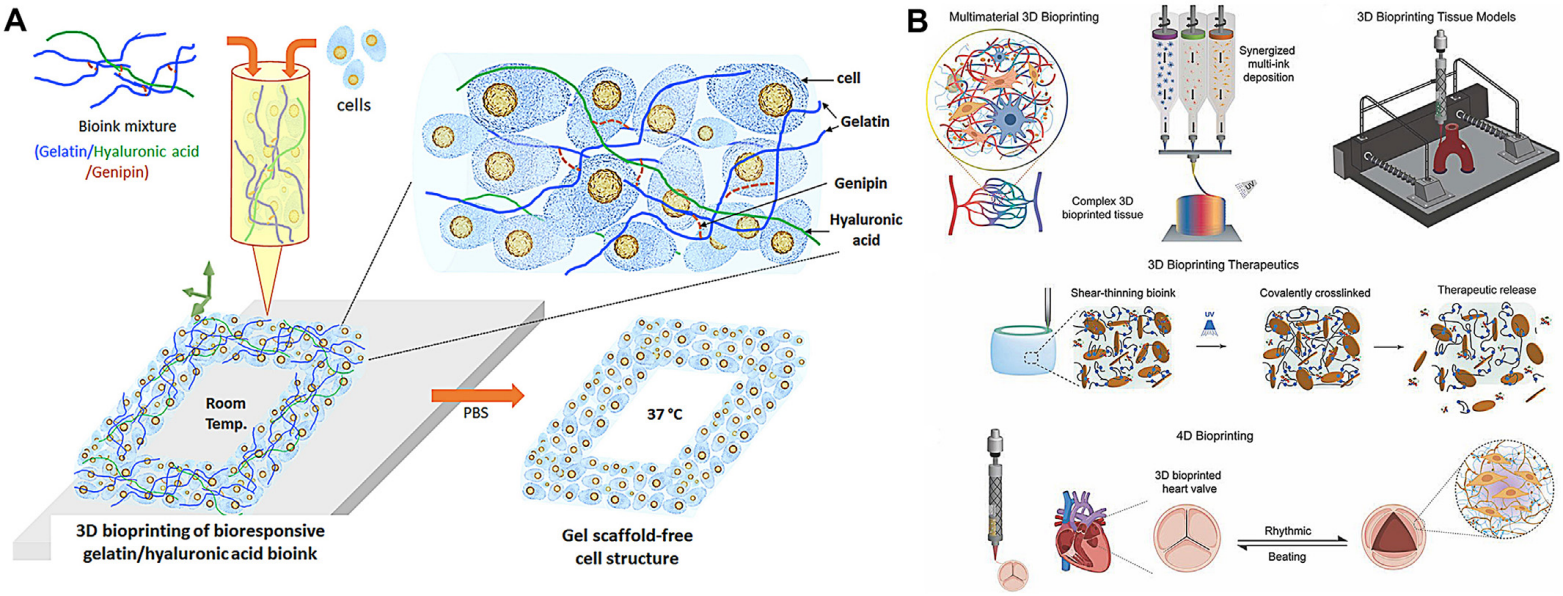
(A) A 3D bioprinting design using a bioresponsive gelatin-hyaluronic acid hydrogel. Copyright permission from Ref. [64]. (B) Multimaterial 3D bioprinting aims to replicate the complicated composition of original tissue structures by synergistically printing various bioinks. 3D bioprinting-engineered tissue models allow for the creation of in-vitro biomimetic tissue models that can be used to better understand disease progression and treatments for diseases such as cancer. 3D bioprinting treatments use bioinks designed with protein therapeutics to direct cell function in the bioprinted construct. Four-dimensional bioprinting allows for the creation of programmable objects with variable behavior and functionality. Bioprinted heart valve tissue responds to electrical impulses created by cardiac cells, contracts, and expands rhythmically. Copyright permission from Ref. [65].

Scaffold materials used for 3D bioprinting are divided into natural and synthetic materials. Materials should be selected reasonably and processed into bio-ink suitable for printing (Fig. 12). The printed scaffold material can be shaped with specific mechanical properties and delicate structures, and seed cells can adhere to the scaffold. Survival and function are the research hotspots of 3D bioprinting scaffold materials [65].

### 3.3 Implants

In recent years, studies have shown that HA nanoparticles (n-HA) can induce apoptosis in various tumor cells, and their inhibitory effect on cancer cell proliferation is far greater than their inhibitory effect on normal cells [66]. The primary mechanism of n-HA in inducing tumor cell apoptosis has been elucidated; after n-HA treatment, intracellular oxidative stress in tumor cells increases sharply, endoplasmic reticulum function is blocked, and the mitochondrial apoptosis pathway is upregulated [67]. Zhang et al. [68] used laser sintering to prepare a porous titanium alloy stent with a porosity of approximately 65%, a pore diameter of approximately 504 μm, and mechanical properties similar to bone. The porous titanium alloy stent was coated using slurry foaming. After creating the n-HA coating, it was applied to the rabbit femoral tumor resection defect model. According to the results, porous titanium alloy scaffolds loaded with n-HA can effectively stop tumor metastasis. The n-HA coating stops tumor growth while encouraging the growth of new bone within the pores of the porous titanium alloy scaffolds. However, this model is an animal model of bone tumors, which differs from that of humans, and there is a lack of comparative studies on intravenous administration routes.

### 3.4 Transdermal

The transdermal drug delivery system allows drugs to pass through the skin constantly. Compared with oral preparations, it has advantages in absorption and distribution in the body and high bioavailability, and it is a common external dosage form. At the same time, shortcomings such as poor drug release effects and a lack of skin barrier penetration seriously restrict the development of transdermal drug delivery systems [69]. As an emerging pharmaceutical technology, 3D printing can make drugs have unique delivery characteristics through computer-aided design software, which is superior to traditional pharmaceutical processes, and its combination with transdermal drug delivery systems is a current research hotspot [70]. Nafiseh Elahpour et al. [71] reviewed studies on 3D printing in the field of transdermal drug delivery and the latest studies on wound excipients, orodispersible films, microneedles, and suppositories, providing a reference for on-demand customization and clinical personalized drug delivery.

Microneedle (MN) technology holds great potential for controlled drug delivery, which has garnered attention from researchers and clinics. MNs can penetrate the stratum corneum of the skin and enter the epidermis, avoiding interactions with nerve fibers. MN patches have been manufactured using various types of materials and application processes. Recently, 3D printing has provided flexibility in prototyping and manufacturing methods to produce MN patches with high shape complexity and reproducibility in a one-step manner. We have studied the final success of using iontophoresis-driven nanoparticle MN patches based on 3D printing. After evaluating various types of MNs and manufacturing technologies, we will investigate different 3D printing methods applied to MN patch manufacturing [72].

## 4 3D-printed nano drug performance and challenges

One of the significant challenges in nano drugs is the need to recreate the complex structures of native drugs, which often have complex hierarchical structures with physical features ranging from nanometers to millimeters in size. Although traditional 3D printing technology can generate macroscopic structures, achieving the necessary resolution and fidelity at the nanoscale is often challenging [73]. In contrast, nanoscale 3D printing employs sophisticated materials and manufacturing methods to produce structures with nanoscale precision. By tailoring the properties of printed materials, researchers can create customized medicine that promotes specific cellular responses such as adhesion, proliferation, and differentiation [74]. In addition to its pharmaceutical applications, nanoscale 3D printing is expected to be used to develop advanced drug delivery systems and diagnostic tools. Nanoscale 3D printing can fabricate microfluidic devices and biosensors with high resolution and sensitivity, allowing for the rapid detection and monitoring of biomarkers and other diagnostic indicators [75]. Although nanoscale 3D printing has great potential, certain challenges must be solved before it can be widely used in pharmaceutical and regenerative medicine. One of the major obstacles is the development of biocompatible materials that can be printed with high resolution and fidelity while providing the necessary mechanical and biochemical cues to support drug delivery [76].

Additionally, the scalability of nanoscale 3D printing remains an issue, as many current technologies are limited in the size and complexity of structures that can be produced. Finally, integrating chemical networks and other essential components into printed medicine remains a significant challenge, as these features are critical for survival and function. A novel frontier in drug delivery systems, 3D-printed nanoparticle medications provide a personalized method of drug distribution. 3D printing technology is used to precisely build these medications, enabling the creation of complex structures suited to certain therapeutic requirements. The capacity to design intricate geometries improves the effectiveness of drugs by permitting targeted distribution to particular bodily locations. The ability of 3D-printed nanomedicines to enhance patient outcomes and compliance is a key benefit. The adaptable nature of these medications enables customized treatment regimens, dosage modifications, and release patterns according to the needs of each patient. This customized strategy may result in fewer adverse effects, more effective therapies, and improved patient well-being all around. By overcoming current challenges and limitations, nanoscale 3D printing has the potential to transform the field of pharmaceuticals in a new era of regenerative medicine [77,78].

**Fig. 13:**
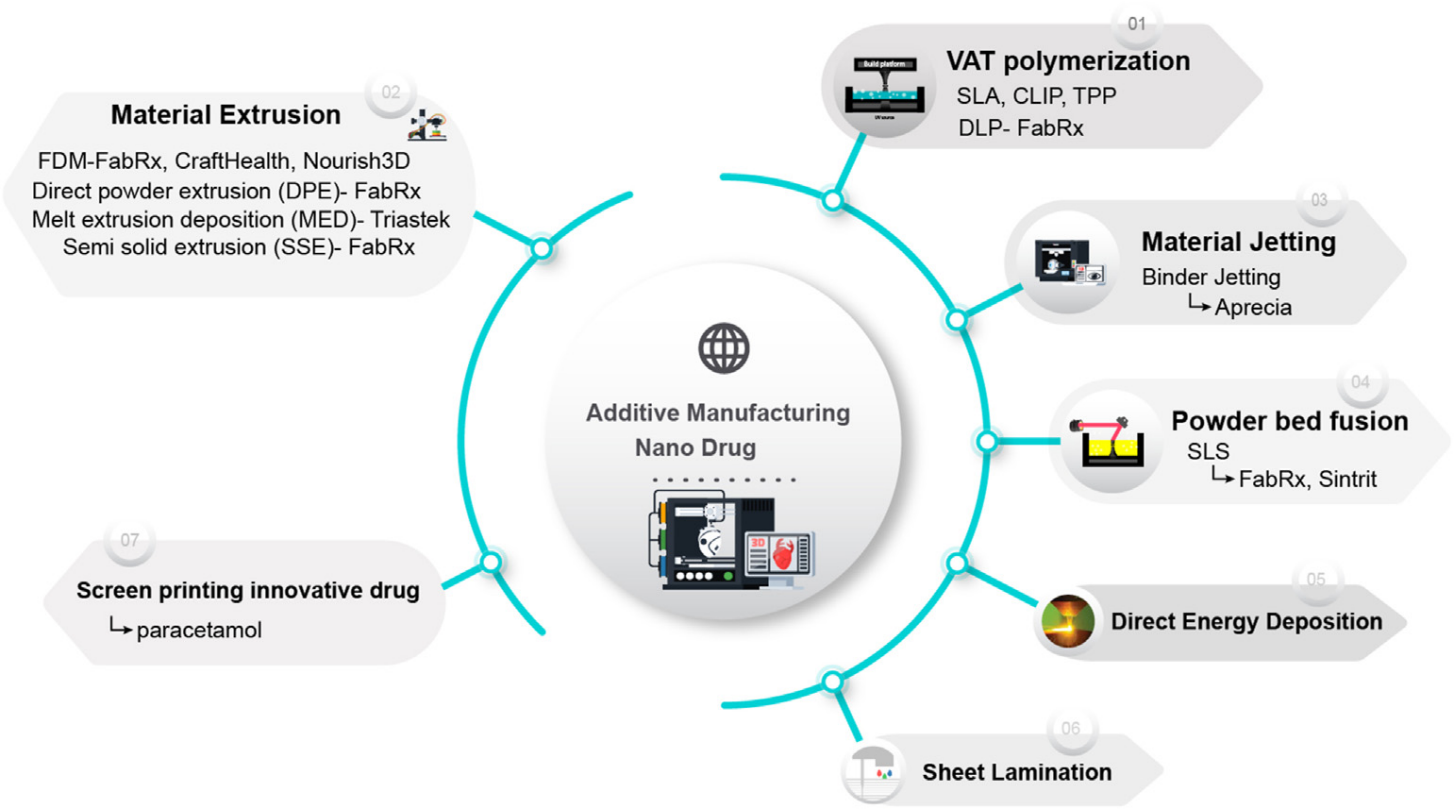
Illustration of 3D printed technologies and 3D printing technology applied to pharmaceutical product development and commercialization.

Compared with traditional preparations, 3D printing preparations are highly controllable and flexible. Controllability is reflected explicitly in controlling complex spatial structures, precise preparation processes, flexible dosage, demand, and structure. 3D printing preparations can be made with multiple release mechanisms by controlling the external shape and internal structure of the preparation and changing the presence and distribution of the drug. 3D printing can realize personalized dosage control, which is suitable for the administration of particular groups or drugs with high toxicity and a narrow therapeutic window; 3D printing can be used according to the patient's gender, age, and weight, among other physiological factors [79]. Based on the principles of powder-liquid bonding and layer-by-layer superposition, 3D printing can achieve the preparation of high drug loading by reducing the number of medication times and the dosage of a single dose, thereby significantly improving patient compliance. Extremely low-dose products have been prepared using 3D printing technology, with specifications as low as 3 ng and an RSD of only 10%, indicating that 3D printing technology is promising for preparing extremely low-dose products that are difficult to achieve with traditional processes [80]. 3D printing has broad prospects in children's medication. In addition to enabling personalized dosage administration for children, 3D printing can precisely control the appearance of tablets and print cartoon preparations with unique shapes and colors, improving clinical compliance with children's medicine [81,82]. Finally, the powder-liquid-bonded 3D printing material is exposed to space for a long period, resulting in the long-term stability of the powder. Recycling is an issue worthy of attention and resolution. Compared with traditional preparations, the current high cost of 3D printing preparations has become an essential factor restricting their development. In 3D-printed pharmaceuticals, around seven major production procedures are used. Startups operating in the field are listed adjacent to the process types in Fig. 13 below. Laxxon Medical recently introduced a new 3D printing process, 3D screen printing, to the market with the brand Screen Printing Innovative Drug. However, there are several challenges to the broad use of 3D-printed nanoparticle medications. A barrier exists to translating promising laboratory data to clinical trials, which calls for thorough testing and validation procedures. Another crucial issue is ensuring that production can grow while still upholding uniformity and quality control. Furthermore, to achieve safety and efficacy standards, these novel drug delivery systems must carefully navigate an ever-evolving regulatory landscape. Researchers, physicians, and regulatory agencies must work together to address these issues. Materials science, 3D printing technology, and a lot of preclinical and clinical research are needed for 3D-printed nanomedicines to fully live up to their promise of changing how medicines are delivered and improving patient outcomes [83].

**Fig. 14:**
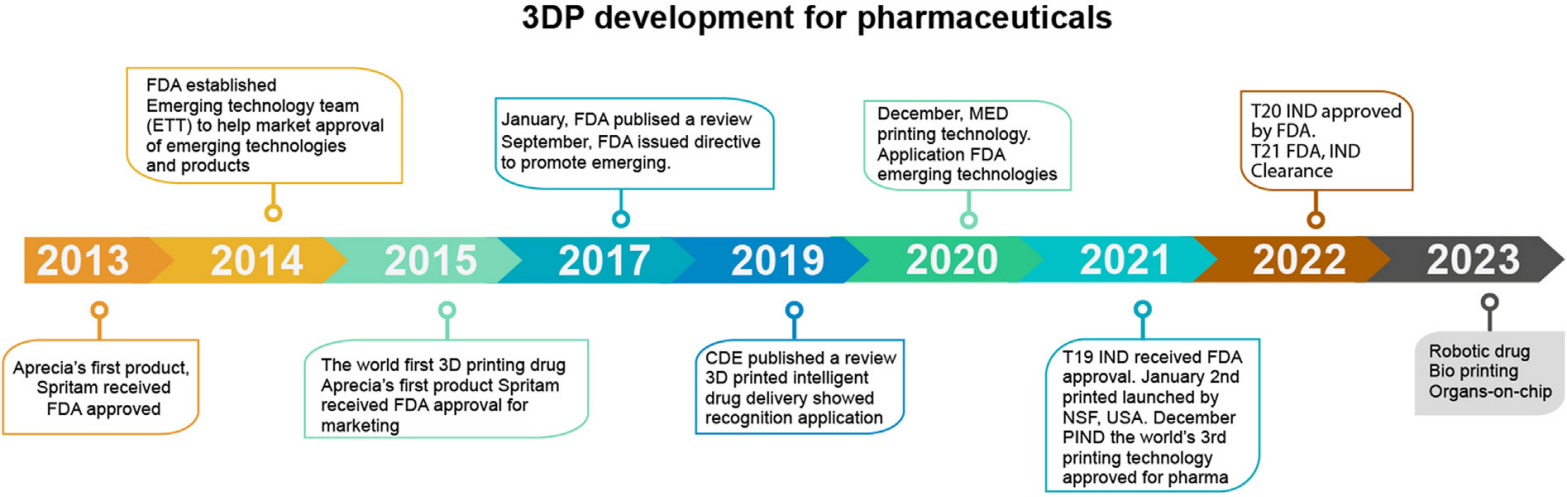
Illustration of the development timeline of the 3D printing process for pharmaceuticals.

Following the current drug production model, in line with the current laws of drug development, registration, and commercial circulation, 3D printing technology has successively developed fixed-dose pharmaceutical products, carried out drug registration and large-scale production, and supplied markets to various countries. Pharmaceutical 3D printing professional companies Aprecia of the United States and Triassic of China have moved forward in this direction and applied 3D printing technology to pharmaceutical product development and commercialization stages. Aprecia has developed a large-scale production system that meets GMP requirements, can achieve drug production of 100,000 tablets per day, and has already launched a 3D-printed drug. Triassic has an automated and continuous GMP 3D printing production line with an annual production capacity of 50 million pieces. Two drugs, T19 and T20, have received the FDA's clinical trial (IND) approval (Fig. 14). In addition, Merck, a large multinational pharmaceutical company, is exploring the direction of large-scale production and has launched an innovative drug 3D printing project. Currently, it uses drug 3D printing technology to produce clinical trial drugs and plans to use it for large-scale production. Based on the data, it is predicted that in clinical phases I–III, the preparation and development time will be reduced by 60%, and the raw materials required to prepare the drug will be reduced by 50% [[84], [85], [86]].

Pharmaceutical 3D printing is the next-generation technology with the greatest potential to change drug manufacturing, but it still faces significant challenges in development and application. Regarding technology development, although there are currently a variety of commercial 3D printers on the market, most of them are difficult to “migrate” directly to pharmaceuticals. Moreover, it is necessary to start from scratch and develop special equipment to meet pharmaceutical requirements and drug regulations, conduct excipient research on pharmaceutical processes and dosage form design, and conduct in vivo and in vitro research and verification on the release mechanism of new dosage forms with three-dimensional drug structures. The overall development of technology is complex and requires high personnel requirements, requiring the full cooperation of talents from many professional disciplines such as engineering, materials science, and pharmacy [87].

Regarding technology application, as 3D printed drugs use brand-new pharmaceutical technology, pharmaceutical 3D printing companies need to go through the regulatory paths of specific countries to ensure the future commercialization of products. For regulators, there is a need to adapt and accept 3D printing as a way to manufacture drugs and be prepared for the changes brought by new technologies. The continuous and digital production process of pharmaceutical 3D printing is the direction of industrial reform promoted by regulatory authorities in various countries. In 2017, the FDA issued industry guidelines to promote the use of emerging technologies in pharmaceutical innovation, and 3D printing of drugs is one of the strategic directions. In 2022, China CDE accepted the IND application for Triassic product T19, focusing on the application of 3D printing in the pharmaceutical industry [[88], [89], [90]].

## 5 Conclusion and future outlook

The utilization of 3D printing technology in the production of pharmaceutical preparations offers a range of advantages, including enhanced flexibility and controllability. This has led to the emergence of novel concepts and approaches to creating pharmaceuticals, particularly in the screening, development, and manufacturing of nano-drug delivery systems. Consequently, there is significant potential for further advancements in this field. The phenomenon of bonding between a powder and a liquid and the utilization of 3D printing technology can modify the visual characteristics, physical form, and internal composition of the end product. The manipulation of printing materials may achieve this, such as changing the composition and proportion of the printing fluid and adjusting various process parameters. The preparation has the potential to exert improved control over the pace, duration, and mechanism of drug release. Positional drug delivery systems, such as pulse and sustained-control release preparations that align with “chrono pharmacology,” can reduce toxic side effects and increase patient compliance. At the same time, the advantages of personalized drug delivery through 3D printing provide technical support for people to realize personalized medicine. In the future, doctors can issue electronic prescriptions based on the patient's diagnosis and transmit them directly to the pharmacy or outpatient clinic. Pharmacists can prepare on-site prescriptions based on electronic prescriptions and print medications in dosage forms or dosages suitable for that patient to better address complex dosing regimens and the medication needs of special patient populations. 3D printing still faces many constraints and challenges, but they will be gradually solved in the future development process. 3D printing technology will open a new chapter for the research and development of pharmaceutical preparations.

The great promise of additive manufacturing in nano-drug delivery systems is about to usher in a new era of precision medicine. The nanoscale creation of intricate drug carriers made possible by 3D printing technologies makes it possible to deliver medications in a targeted and personalized manner. This novel combination reduces side effects while increasing therapeutic efficacy. We predict the arrival of personalized nanomedicine compositions based on the needs of each patient as improvements continue. The adaptability and scalability of additive manufacturing will expedite production and promote affordable solutions. This convergence of technologies offers previously unheard-of opportunities for highly efficient, patient-centric, and effective medication delivery systems, paving the way for revolutionary advancements in healthcare.

## Credit authorship contribution statement

Md. Habibur Rahman completed the writing task; Nilufar Yasmin Liza completed the literature search and screening; Md. Abu Shyeed wrote and reviewed the manuscript; Dipika Ramdas Kalambhe checked and reviewed the writing quality of the article; Dilwar Hossain Noor oversaw resources and reviewing; Khan Rajib Hossain determined the direction and content composition of the article and checked and reviewed the writing quality of the article. All authors have reviewed and approved the final manuscript, attesting to its accuracy and their collective commitment to the review.

## Data availability

Not applicable.

## Ethics approval

Not applicable.

## Funding information

No agency funding was used in the drafting of this manuscript.

## Declaration of interest

The authors declared that they have no involvement of affiliations with any financial institution or entity with any financial or non-financial interest in the contents discussed in this manuscript. The authors also do not have any conflict of interest.
